# Erlotinib augmentation with dapsone for rash mitigation and increased anti-cancer effectiveness

**DOI:** 10.1186/s40064-015-1441-5

**Published:** 2015-10-23

**Authors:** R. E. Kast

**Affiliations:** IIAIGC Study Center, 22 Church Street, Burlington, VT 05401 USA

**Keywords:** Dapsone, Erlotinib, Glioblastoma, Interleukin-8, Ovarian cancer, Neutrophils, Non-small cell lung cancer, Pancreas cancer, Rash, Quality of life

## Abstract

**Background:**

The epidermal growth factor receptor tyrosine kinase inhibitor erlotinib has failed in many ways to be as potent in the anti-cancer role as pre-clinical studies would have suggested. This paper traces some aspects of this failure to a compensatory erlotinib-mediated increase in interleukin-8. Many other-but not all- cancer chemotherapeutic cytotoxic drugs also provoke a compensatory increase in a malignant clone’s interleukin-8 synthesis. Untreated glioblastoma and other cancer cells themselves natively synthesize interleukin-8. Interleukin-8 has tumor growth promoting, mobility and metastasis formation enhancing, effects as well as pro-angiogenesis effects.

**Findings:**

The old sulfone antibiotic dapsone- one of the very first antibiotics in clinical use- has demonstrated several interleukin-8 system inhibiting actions. Review of these indicates dapsone has potential to augment erlotinib effectiveness. Erlotinib typically gives a rash that has recently been proven to come about via an erlotinib triggered up-regulated keratinocyte interleukin-8 synthesis. The erlotinib rash shares histological features reminiscent of typical neutrophilic dermatoses. Dapsone has an established therapeutic role in current treatment of other neutrophilic dermatoses.

**Conclusion:**

Thus, dapsone has potential to both improve the quality of life in erlotinib treated patients by amelioration of rash as well as to short-circuit a growth-enhancing aspect of erlotinib when used in the anti-cancer role.

## Background

This paper points to a simple drug addition to erlotinib that is planned to make erlotinib both better tolerated and more effective in the anti-cancer role. Erlotinib is a 393 Da tyrosine kinase inhibitor with specific activity for the epidermal growth factor receptor [EGFR] also termed HER-1 (Zahonero and Sanchez-Gomez 2014). Rash, diarrhea, and fatigue are common side effects (Tiseo et al. 2014). The typical erlotinib rash can be quite bothersome in a significant minority of those treated, lowering quality of life (Tiseo et al. 2014). Erlotinib has been shown to prolong overall survival in non-small cell lung cancer (D’Arcangelo and Cappuzzo 2013; Pallis and Syrigos 2013; Zhang et al. 2012) and pancreatic cancer with gemcitabine (Zahonero and Sanchez-Gomez 2014; Park et al. 2013; Vaccaro et al. 2013) or with capecitabine (López et al. 2013), indications for which it is widely used and approved by national regulatory authorities. However, in pre-treated patients median overall survival (OS) is less than a year in both pancreas cancer (Vaccaro et al. 2013; López et al. 2013; Stepanski et al. 2013) and in non-small cell lung cancer (Van Meerbeeck et al. 2014; Kaburagi et al. 2013).

Good tumor suppressive effects in head and neck cancer is typically associated with rash or mucositis in a third of treated patients (Gross et al. 2014). Erlotinib has useful effects like this in several cancers when used “off-label”, for example, muscle invasive bladder cancer can be significantly down-staged after 4 weeks preoperative erlotinib (Pruthi et al. 2010). With erlotinib (plus bevacizumab) in advanced platinum-resistant epithelial ovarian cancer [EOC] 70 % of patients progressed by 6 months (Chambers et al. 2010). Glioblastoma patients treated at first recurrence with erlotinib had a median 10 month overall survival with 20 % of treated patients experiencing severe rash (Yung et al. 2010). When treating recurrent glioblastoma, erlotinib at 150 mg daily gives a 3 month progression-free survival and 9 month overall survival (Peereboom et al. 2010). Recent study of erlotinib with temsirolimus gave no better results (Wen et al. 2014). In selected good-prognosis post-resection, post-radiation (but never chemotherapy) glioblastoma patients, daily erlotinib gave a 3 % non-progression at 6 months and 40 % death at one year (Raizer et al. 2010). Erlotinib monotherapy in recurrent glioblastoma gave a median 7 month OS (Kesavabhotla et al. 2012).

The high incidence of EGFR overexpression in pediatric high grade gliomas (Zahonero and Sanchez-Gomez 2014) provided the rationale for conducting a pediatric study using EGFR inhibitors. The use of erlotinib during and after local radiotherapy did not change the poor outcome of children with intracranial high grade gliomas (Qaddoumi et al. 2014). The combination of erlotinib and rapamycin, an mTOR inhibitor, for recurrent low grade glioma patients was, in general, well tolerated but had questionable to no activity (Yalon et al. 2013). Clearly, erlotinib needs help.

Why erlotinib has not been more effective even in the presence of overexpression of EGFR, remains unclear (Zahonero and Sanchez-Gomez 2014) but a crucial question to answer. This paper now attempts a contribution to that answer.

Cells generally, and cancer cells specifically, are not passive recipients of therapeutic interventions. They react. As in the Preamble, interventions commonly and inherently generate reactions with effect opposing the original intent. In cancer treatment, this means reactions with potential to enhance tumor growth. This paper addresses one such compensatory growth-enhancing reaction- the elevation of interleukin-8 (IL-8) by erlotinib. The reviewed data will show how erlotinib-provoked IL-8 elevation has three negative effects on treatment efforts: (1) decreasing quality of life by generation of the neutrophilic erlotinib rash, (2) IL-8 mediated enhanced migration or invasion of cancer cells in a similar manner as has been shown for neutrophils’ chemotaxis along an IL-8 gradient, and (3) enhanced neutrophil migration to and into tumors with ensuing growth contribution by factors brought to tumors by these neutrophils.

Past research leads to a conclusion that the old anti-Hansen’s disease sulfone antibiotic dapsone, when added to erlotinib, can be expected to mitigate all three IL-8-related erlotinib consequences that currently detract from erlotinib’s anti-cancer effectiveness.

## The erlotinib rash

IL-8 (synonymous with CXCL-8) is an inflammation-mediating, pro-angiogenic, ~8 kDa cytokine/chemokine, signaling via CXCR1 or CXCR2 receptors (Gales et al. 2013; Baggiolini et al. 1995). IL-8 is synthesized by normal cells, particularly keratinocytes, and by dozens of cancer types where it is involved in, and contributes to, several domains of cancer pathophysiology (Gales et al. 2013; Lippitz 2013). IL-8 is central to both normal and pathogenic angiogenesis (Desbaillets et al. 1997).

Development of rash during erlotinib treatment is a common occurrence. Typical erlotinib rashes are pleomorphic, appearing clinically as pruritic or nonpruritic papules, pustules, xerodermia, or paronychia (Kiyohara et al. 2013). In confirming earlier studies of Nardone et al., in 2012, Bangsgaard et al. showed unequivocally that an iatrogenic erlotinib rash experimentally induced in healthy human volunteers was caused by IL-8 mediated neutrophil infiltration (Nardone et al. 2010; Bangsgaard et al. 2012). This would put erlotinib rashes solidly within the neutrophilic dermatoses’ category (Wallach and Vignon-Pennamen 2006)—a category in which dapsone has a long clinical history for being effective (Paniker and Levine 2001; Wozel and Blasum 2014; Cohen 2009).

Depending on dosing and study conditions, erlotinib rash can be seen in 75–90 % of treated patients, being severe in ~10 % (Tan and Chan 2009; Jia et al. 2009; Rozensztajn et al. 2014) This rash is usually treated with topical antibiotics, meticulous skin care, minocycline and topical steroids (Kiyohara et al. 2013). Development of an erlotinib rash confers clear but minor overall survival advantage when treating various cancers (Stepanski et al. 2013; Kaburagi et al. 2013; Peereboom et al. 2010; Kiyohara et al. 2013; Aranda et al. 2012; Petrelli et al. 2012; Fiala et al. 2013) with some indication that greater rash severity is correlated with slightly longer OS than lesser severity rashes (Kiyohara et al. 2013; Rozensztajn et al. 2014; Petrelli et al. 2012) A statistically significant association exists between higher erlotinib levels and greater likelihood of rash (Tiseo et al. 2014; Fukudo et al. 2013).

Erlotinib is not unique in stimulating IL-8 synthesis- many cancer chemotherapeutic drugs do. Urothelium exposed to doxorubicin at levels used in intravesical treatment triggered IL-8 synthesis (Kang et al. 2013). Paclitaxel and temozolomide doubled IL-8 synthesis in melanoma cells (Luo et al. 2012). 5-Fluorouracil (5-FU) induced IL-8 upregulation in prostate cancer cells, an effect mediated by IL-8 signaling at CXCR2 (Wilson et al. 2008a). Most importantly for the proposed role for dapsone, in that study direct 5-FU cytotoxicity reduction by that compensatory IL-8 increase could be mitigated by an experimental CXCR2 blocker (Wilson et al. 2008b), an effect expected to be duplicated by dapsone. Oxaliplatin induced increased IL-8 in metastatic prostate cells (Wilson et al. 2008a). Irradiation, 5-FU and cisplatin each individually triggered an increase in IL-8 in head and neck cancer cell lines (Reers et al. 2013). Topotecan increased IL-8 in breast cancer (Wan et al. 2012). Melanoma cells naturally secrete abnormally high levels of IL-8, levels that further increase after exposure to etoposide or doxorubicin (Merighi et al. 2009). Even the surgery of EOC resection can trigger systemic increase in IL-8 (Dong et al. 2012). It is reasonable to assume that, were dapsone to indeed decrease IL-8 signaling, and its consequences, during erlotinib treatment, dapsone could find wide applicability in augmenting other cancer chemotherapies that engage compensatory increases in IL-8.

Thus, elevation of IL-8 seems to be a common-but not universal- attribute of cytotoxic chemotherapy drugs. Why rash is more common with erlotinib remains unexplained. IL-8 elevations in untreated cancer and its further elevation by cancer chemotherapies are examples of the Preamble chess aphorism that indeed apply to medicine.

## Dapsone

Dapsone is a 248 Da antibiotic introduced into clinical practice in the late 1940s and still used worldwide. Dapsone is active against, and used clinically to treat various species of Pneumocystis, Plasmodia, Toxoplasma, and Mycobacteria (Paniker and Levine 2001; Coleman 1993. Dapsone level is typically 2–5 microg/mL serum after single dose (Opravil et al. 1994; Swain et al. 1983). Time to Cmax is ~4 h, T ½ about 30 h, with significant variability and enterohepatic circulation (Zuidema et al. 1986). Therapeutic serum levels are thought to be between 0.5 and 5 microg/mL when used in antibiotic and anti-protozoa roles (Zuidema et al. 1986). Optimal blood levels when treating neutrophilic dermatoses have not been determined. CNS penetration of dapsone is excellent, cerebrospinal fluid levels reaching 50–100 % of serum levels (Rich and Mirochnick 1996), a particularly valuable attribute when treating glioblastoma. Although most sulfonamide allergic patients tolerate dapsone without problem, dapsone inhibits dihydrofolic acid synthesis by competing with para-aminobenzoic acid for the active site of dihydropteroate synthetase, preventing conversion of para-aminobenzoic acid to dihydrofolic acid (Coleman 1993) as do sulfonamides.

Independently of its antibiotic attributes, dapsone is active in treating autoimmune bullous diseases (Piette and Werth 2012) and neutrophilic dermatoses (Paniker and Levine 2001; Wozel and Blasum 2014; Cohen 2009). In bullous pemphigoid dapsone stops bullae formation and often the rash but does not affect the underlying sub-epidermal auto-antibody fixation (Booth et al. 1992; Kasperkiewicz and Zillikens 2007; Shimanovich et al. 2004). But dapsone does stop the consequent IL-8 mediated recruitment of neutrophils to areas of autoantibody deposition. Thereby dapsone stops bullae formation (Booth et al. 1992; Kasperkiewicz and Zillikens 2007; Shimanovich et al. 2004). Given the work of Nardone et al. and of Bangsgaard et al. quoted above (Nardone et al. 2010; Bangsgaard et al. 2012) and the action of dapsone in autoimmune bullous diseases, in like manner dapsone will mitigate erlotinib rashes.

Of crucial importance to this intended use, dapsone inhibits LPS stimulated IL-8 production in human keratinocytes (Schmidt et al. 2001). IL-8 synthesis was also reduced both in LPS stimulated bronchial epithelial cells in vitro (Kanoh et al. 2011), and in LPS stimulated peripheral blood mononuclear cells (Abe et al. 2008). Dapsone quantitatively reduced mRNA expression of endothelin-1 (ET-1), macrophage inflammatory protein-1 alpha (MIP-1 alpha), and transforming growth factor-beta (TGF-beta) in a mouse model of paraquat lung injury (Cho et al. 2011). ET-1 (Kast 2009; Liu et al. 2011; Patel and McKeage 2014; Paolillo et al. 2010), MIP-1 (Fang et al. 2011; Weigert et al. 2009 and TGF-beta (Hau et al. 2011; Joseph et al. 2013) are, all three to varying degrees, shown to contribute to many cancers’ growth and treatment resistance, including glioblastoma (Fang et al. 2011; Stiles et al. 1997) and EOC (Kast 2009; Vergara et al. 2010). A crucial question for determining dapsone’s potential in the anti-cancer role would be to determine if these mRNA reductions are a primary effect of dapsone or a secondary effect to action at a paraquat-specific trigger to these mRNA elevations.

Dapsone has already been suggested as an adjunct to glioblastoma treatment by blocking IL-8 directed migration of neutrophils into tumor, thereby lowering neutrophil-delivered VEGF to a growing glioblastoma (Kast et al. 2011, 2012). As an ancillary benefit, by inhibiting 5-lipoxygenase (5-LOX), dapsone diminishes leukotriene synthesis (Wozel and Blasum 2014; Wozel and Lehmann 1995). 5-LOX products contribute to growth in glioblastoma (Morin et al. 2013; Ishii et al. 2009) and other cancers (Bishayee and Khuda-Bukhsh 2013; Meng et al. 2013; Wen et al. 2015).

## IL-8

IL-8 upregulation is a native feature of many cancers, forming an active growth facilitating factor for these cancers (Gales et al. 2013; Shi et al. 2001; Campbell et al. 2013). Yet more central to cancer physiology, IL-8 forms a stronger growth enhancing role in the stem sub-population than in the non-stem population in breast cancer (Singh et al. 2013a, 2013b) hepatocellular carcinoma (Tang et al. 2012), squamous head/neck cancer (Chikamatsu et al. 2011), glioblastoma, (Infanger et al. 2013; Bonavia et al. 2012) and others.

Exogenous IL-8 stimulated EOC cell line proliferation in vitro (Wang et al. 2005, 2011; Merritt et al. 2008; Wang et al. 2012). By immunohistochemistry only half of EOC patient biopsies were positive for high IL-8 expression, with higher expression correlating with shorter overall survival (Merritt et al. 2008). Although confirming EOC synthesis of IL-8 using immunohistochemistry of EOC surgical resection tissue, others found no relationship between staining intensity and OS (Browne et al. 2013).

By in vitro hybridization in EOC biopsies IL-8 mRNA was found to be overexpressed (Davidson et al. 2003). EOC cell lines synthesize and secrete IL-8, with more IL-8 being synthesized in estrogen receptor negative cell lines, less in estrogen receptor positive lines (Davidson et al. 2003). Serum IL-8 levels were higher in EOC than in controls (Lokshin et al. 2006; Autelitano et al. 2012), with higher levels predicting shorter OS (Bertenshaw et al. 2008; Dobrzycka et al. 2013; Kassim et al. 2004). EOC tumor associated macrophages have the ability to stimulate EOC cells’ IL-8 synthesis (Wang et al. 2013). EOC migration and invasion is stimulated by omental adipocytes, an effect largely mediated by IL-8 (Nieman et al. 2011).

EOC cell migration along an IL-8 gradient (Ma et al. 2011) is reminiscent of similar findings of neutrophil chemotaxis along an IL-8 gradient. Since solid data shows dapsone blinding of neutrophils’ migration along an IL-8 gradient, similar blinding by dapsone of EOC cells can be expected. IL-8 figures prominently in the typical intense neovascularization in EOC (Yang et al. 2010).

In vitro EOC resistance to cisplatin and paclitaxel is enhanced by exogenous IL-8 (Wang et al. 2011). Epinephrine or norepinephrine exposure resulted in more than doubling of IL-8 protein and mRNA in EOC cells, with even greater increases in the IL-8 promoter, effects that were all blocked by propranolol (Shahzad et al. 2010).

The importance of Cho et al’s demonstration of TGF-beta mRNA decreases after dapsone (Cho et al. 2011) meshes nicely with Serizawa et al’s demonstration of enhancement of erlotinib’s effects on non-small cell lung cancer if TGF-beta is simultaneously inhibited (Serizawa et al. 2013). This also supports a trial of dapsone augmentation of erlotinib.

Adding to the appeal of using dapsone to retard neutrophil migration to cancers are two further aspects. One: In two underappreciated but truly elegant papers, Werther et al. (2002) and Svensen et al. (2004) proved that circulating neutrophils deliver VEGF to rectal cancers. By measuring neutrophil number and VEGF content in the arterial supply and in the venous drainage of these tumors, they found 16 % of VEGF containing neutrophils entering the tumor do not exit it (Werther et al. 2002; Svendsen et al. 2004). Also neutrophils are not just a source of VEGF (Kast et al. 2011, 2012; García-Román and Zentella-Dehesa 2013; Lee et al. 2002) and IL-8- they deliver other growth factors too.

Neutrophils are a prodigious source of multiple other cytokines and growth factors of importance in promoting cancer’s growth and treatment resistance (Lee et al. 2002; Scapini et al. 2000; Kasama et al. 2005). Two: During both normal and pathological angiogenesis endothelial IL-8 and neutrophils’ IL-8 have an interesting relationship. When endothelium is exposed to IL-8 secreted by neutrophils this awakens and stimulates vessel endothelium to synthesize IL-8, generating a stronger IL-8 gradient along which neutrophils home, bringing yet more IL-8 to the site, thus forming a feedforward amplification cycle first clearly identified by Garcia-Roman et al. in 2002 (García-Román and Zentella-Dehesa 2013) and (Schruefer et al. 2005).

IL-8 synthesized by keratinocytes after minor wounds attracts neutrophils to the wound (Lan et al. 2013). High ambient glucose, as obtains in diabetes, increases such IL-8 mediated neutrophil migration to wound when neutrophil over-population delays rather than hastens wound healing (Lan et al. 2013). Dapsone normalized wound healing in a diabetic rat model by reducing such excess neutrophil accumulation (Lan et al. 2013).

A little noted breakthrough in understanding glioblastoma pathophysiology came from a study showing that endothelial cells can upregulate their synthesis of IL-8 in response to glioblastoma cells’ secreted IL-8, and vice versa (Infanger et al. 2013), findings that replicate the feedforward system of Schruefer et al. (2005) between neutrophils and endothelial cells, findings that again support the potential role for dapsone in glioblastoma treatment.

Glioblastoma (Infanger et al. 2013; Zhang et al. 2015), non-small cell lung cancer (Liu et al. 2015; Khan et al. (2015), ovarian cancer (Wang et al. 2011; Browne et al. 2013; Stronach et al. 2015) and many other cancers’ (Sanmamed et al. 2014) cells synthesize IL-8 and express its receptor(s). IL-8 has specifically been recognized as a factor mediating erlotinib resistance in non-small cell lung cancer (Liu et al. 2015). As Zhang et al. suggest, IL-8 would be a “novel therapeutic target for glioma invasion intervention” (Zhang et al. 2015). Dapsone may provide such, resurrecting erlotinib usefulness for glioblastoma and other cancers.

## Caveats

Erlotinib absorption is facilitated by gastric acid so use of proton pump inhibitors should be avoided. Tobacco use limits erlotinib absorption so use of all tobacco products must be stopped. Dapsone’s primary metabolite N-hydroxydapsone inhibits leukotriene chemotaxis and has other anti-inflammatory attributes (Wozel et al. 1997) of potential benefit during cancer treatment, but not further discussed here.

## Conclusions

This paper reviewed the growth promoting aspects of IL-8 in a few selected cancers and data showing how erlotinib, a drug designed to- and in a limited way succeeded in decreasing cancer growth, can and often does increase IL-8 in skin. The data on neutrophils’ sine qua non role in generating the erlotinib rash implies a rash-mitigating role for dapsone. During clinical use in the neutrophilic dermatoses like bullous pemphigoid, dapsone blinds neutrophils to IL-8 chemotaxis, suggesting by extension that dapsone can ameliorate erlotinib rash, increasing tolerability and quality of life. The tumor growth enhancing effects of IL-8 itself and via neutrophil recruitment to a growing tumor were reviewed. Effects expected to be partially thwarted by dapsone.

Perhaps the most pivotal, telling, and dramatic insight into erlotinib action, and of particular relevance to dapsone augmentation is the recent work of Takashima et al. (Takashima et al. 2012). A cohort of 76 erlotinib-treated (150 mg/day) advanced, previously treated non-small cell lung cancer patients developing a rash were divided into two groups—24 whose erlotinib dose was down-titrated to rash mitigation and 31 who were left at the rash-generating dose. Median OS was 566 days in the dose reduction, rash mitigation group but 202 days in the dose maintained, original dose group (Takashima et al. 2012). Such a result would seem to contradict the many studies showing rash as a marginally favorable sign. If dose-related rash mitigation and longer OS is the result of decreasing compensatory IL-8 drive secondary to decreased erlotinib dose as the reviewed data suggest, then dapsone augmentation may well work by similarly decreasing IL-8 related growth drive and give erlotinib help it clearly needs (Zahonero and Sanchez-Gomez 2014).

## Abbreviations

ET-1: endothelin-1
EGFR: epidermal growth factor receptor
EOC: epithelial ovarian cancer
IL-8: interleukin-8 (synonymous with CXCL8)
OS: overall survival
TGF-beta: transforming growth factor-beta
VEGF: vascular endothelial growth factor
